# Malaria caused by *Plasmodium vivax*: recurrent, difficult to treat, disabling, and threatening to life — averting the infectious bite preempts these hazards

**DOI:** 10.1179/2047772413Z.000000000179

**Published:** 2013-12

**Authors:** J Kevin Baird

**Affiliations:** 1 Eijkman-Oxford Clinical Research Unit, Jakarta, Indonesia; 2 The Centre for Tropical Medicine, Nuffield Department of Medicine, University of Oxford, UK

**Keywords:** *Plasmodium vivax*, severity, difficulty of therapy, prevention, vector control

## Abstract

The maxim ‘*an ounce of prevention is worth a pound of cure*’ finds few better demonstrations than with malaria caused by *Plasmodium vivax*. Thoroughly neglected over the past 60 years, the chemotherapy of this complex infection has been dangerous and ineffective until the present. Work is at last being done, but seeing that translate to real improvements at the periphery of care delivery will take years of deliberate effort. In the meantime, patients face substantial risk of debilitating, threatening, and fatal courses of illness associated with a diagnosis of vivax malaria. For some of the most vulnerable to such outcomes — pregnant women and infants — repeated attacks of acute vivax malaria from a single infectious anopheline bite is now not preventable. One of the few measures than can be immediately applied with rigor is vector control, thereby effectively preventing as many of these difficult and dangerous infections as possible. This commentary emphasizes the dire consequences of infection by *P. vivax* and the real difficulty of dealing with them. That, in turn, emphasizes the many benefits to be derived by preventing them in the first place.

## Onerous Burden

**Figure 1:**
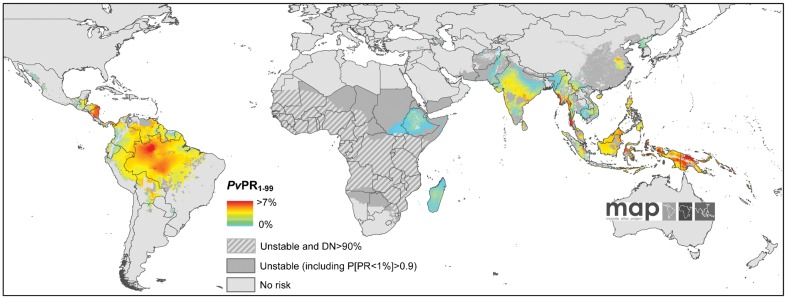
Global distribution of endemic *Plasmodium vivax* (from Ref. 1, published under Creative Commons License).

*Plasmodium vivax* occurs all across the globe, from the Korean Peninsula to the northern fringes of Argentina (Fig. 1^1^). The exact number of annual clinical attacks has not been reliably estimated, but it is thought to be more than 100 and less than 400 million.^2^ That number, whatever it really happens to be, is likely of the same order of magnitude (low hundred millions) as the estimated number of clinical attacks of falciparum malaria, i.e. 247 million according to the World Health Organization,^3^ or 451 million in according to Hay and colleagues.^4^ Nonetheless, malariologists have historically dismissed the heavy burdens of *P. vivax* in Asia and the Americas in weighing the global malaria problem — largely by supposing the relative harmlessness of vivax malaria. Most have thus considered the global malaria problem to be heavily weighted upon a single continent by a single species (*Plasmodium falciparum*; Africa). However, the supposition of harmlessness in vivax malaria does stand to available evidence^5^ and the infection must be combatted with much more rigor than has been applied in the past.

## Biological Complexity

The life cycles of all of the five species of *Plasmodium* naturally infecting humans are complex, but *P. vivax* (and *P. ovale*) has a single additional stage that hugely magnifies its epidemiological and clinical complexity. This parasite places dormant forms in the liver that cause multiple clinical attacks (called relapses) over about 2 years. In contrast, a single infectious bite with *P. falciparum* causes a single attack within less than 2 weeks. The risk, timing, and frequency of these clinical attacks emanating from the liver appears evolved to coincide with both a relatively high likelihood of encountering a different strain of *P. vivax* in the blood^6^ and of the presence of feeding anopheline mosquitoes in the environment (in which sexual recombination with that other strain may occur).

The Chesson and North Korea strains of *P. vivax* represent the two extremes of relapse behaviors. Chesson strain came from an American soldier infected in New Guinea during the Pacific War.^7^ Perpetually tropical and rainy New Guinea has a virtually uninterrupted abundance of anophelines, and Chesson strain relapses very often (virtually 100%), very quickly (within 3 weeks of the primary parasitemia becoming patent), and multiple times (5 is typical, 15 is known) within 2 years (exceptionally 3 years). North Korea on the other hand, has a fleeting summer period where anopheline mosquitoes will occur, and North Korean *P. vivax* strain exhibits no primary parasitemia, but will relapse only 8–12 months after inoculation.^8^

Shute and colleagues^8^ discovered that populations of sporozoites of North Korean *P. vivax* consisted of two types — those that immediately went into full development of liver and blood stages, and those that became the dormant forms responsible for relapse — called hypnozoites. The latter greatly outnumbered the former in that temperate strain, demonstrated by primary parasitemias after inoculating relatively vast numbers of sporozoites. This ratio presumably represents adaptation to the brevity of seasonal anopheline abundance. The parasites emerge into the relative danger of the bloodstream only when the likelihood of encountering feeding anophelines is relatively high. Each such sortie provokes the human misery known as malaria.

## Force of Infection

The hypnozoite impels departure from convention when considering terminology as basic as ‘infection’. The biology of falciparum malaria permits classifying an infectious bite seeding the liver with sporozoites, and subsequent seeding of the blood by merozoites as a single infectious event, i.e. an ‘infection’. If one thinks of the defining event of malaria as the presence of the disease-causing asexual stages in the blood, then the term in vivax malaria takes on different meaning. While hosts harboring quiescent hypnozoites are indeed ‘infected’, there is no active blood infection and therefore, no acute malaria. In this sense, both mosquitoes and hypnozoites in the liver may be considered contributors to the force of infection of the blood.

The liver reservoir of *P. vivax* in any given endemic community may indeed be substantial. While it is now not possible to diagnose latent liver stages, one can, under some circumstances, observe their force of infection. One such study reported by Douglas and colleagues^9^ reported that among hundreds of patients diagnosed with acute falciparum malaria in western Thailand and treated with a rapidly eliminated therapy (rather than one that lingers in the blood for weeks, suppressing new parasitemias from any source), 51% suffered a relapse of *P. vivax* after just 2 months. That number may be considered an absolutely minimal estimation of the prevalence of hypnozoites among people having falciparum malaria (longer follow-up would almost certainly have increased that number). Another study reveals the force of infection by hypnozoites: Downs *et al.*^10^ described malaria attack rates in the US Army Americal Division engaged in combat on Guadalcanal in the Solomon Islands of 1942 and 1943. While on that malarious island, the attack rate was about 1 infection/person-year, despite chemophrophyaxis (using atebrin, a chloroquine-like drug). After several months of heavy combat, the division was evacuated (with mass treatment against blood but not liver stages of malaria) for rest and recuperation at Fiji, where no anopheline mosquitoes occur and malaria transmission is not possible. The malaria attack rates soared to 4 infections/person-year on Fiji — almost certainly all representing activation of hypnozoites.

In some communities, the hypnozoite probably represents the more common origin of a new blood infection event. No study has yet measured both compartments of infection simultaneously. Several studies in Indonesian Papua, where malaria is very heavily endemic, consistently showed incidence density of new infection among subjects (cleared of blood and liver stage parasites for *P. vivax*) at about 2 infections/person-year.^11^ This was considered to be the force of infection from mosquitoes, i.e. sporozoite-borne primary attacks. If each such inoculation then yielded a modest five infections of blood by activation of hypnozoites, its force of infection would of course greatly exceed that number for sporozoites. Such may not be universally true and will depend upon relapse behaviors of local strains.

## A Threatening Event

Although infection by *P. vivax* has long been known as ‘benign tertian malaria’, that dogma of harmlessness has been revealed as fallacious.^2^,^5^ Among patients hospitalized with a primary diagnosis of vivax malaria, about 10–20% will be classified as having severe illness, and 5%–15% of those will not survive. These frequencies approximate those with a diagnosis of malaria caused by *P. falciparum*. The syndromes of severe illness are also broadly similar. Lança *et al.*^12^ found that the WHO criteria for severe falciparum malaria (statistically linked to high risk of a fatal outcome^13^) correlated very well with risk of admission to intensive care units in Brazil with a diagnosis of *P. vivax* malaria. The lone exception was count of parasitemia considered threatening: >250 000/μl for *P. falciparum*, whereas in hospitals in Brazil, patients with *P. vivax* parasitemia >500/μl were often referred to intensive care units. Thus, despite seemingly non-threatening levels of parasitemia in vivax malaria, patients become seriously and sometimes fatally ill.

## Difficult to Treat

Treatment of acute vivax malaria requires two distinct classes of drugs — blood schizontocide(s) (to terminate the acute attack), and hypnozoitocide(s) (to prevent subsequent acute attacks). The two classes compose what is referred to in malariology as ‘radical cure’. This partnership comes with a list of therapeutic issues regarding drug–drug interactions impacting safety or efficacy. Since chloroquine and primaquine were partnered in this capacity in 1952, no other pairs of drugs for radical cure of vivax have been demonstrated safe and effective (excepting quinine and primaquine, also validated during the work with chloroquine). Resistance to chloroquine by *P. vivax* has been emerging at least since 1990 and worsens and spreads from its epicenter in eastern Indonesia.^14^ Though there are many new blood schizontocides with good efficacy against chloroquine-resistant *P. vivax*,^15^ only one has been demonstrated to come with good safety and efficacy against relapse when partnered with primaquine (albeit with dosing delayed by 25 days after treating the acute attack due to safety concerns).^16^ The difficulty of such evaluations will certainly limit therapeutic options for radical cure of vivax malaria with new blood schizontocides.

**Figure 2:**
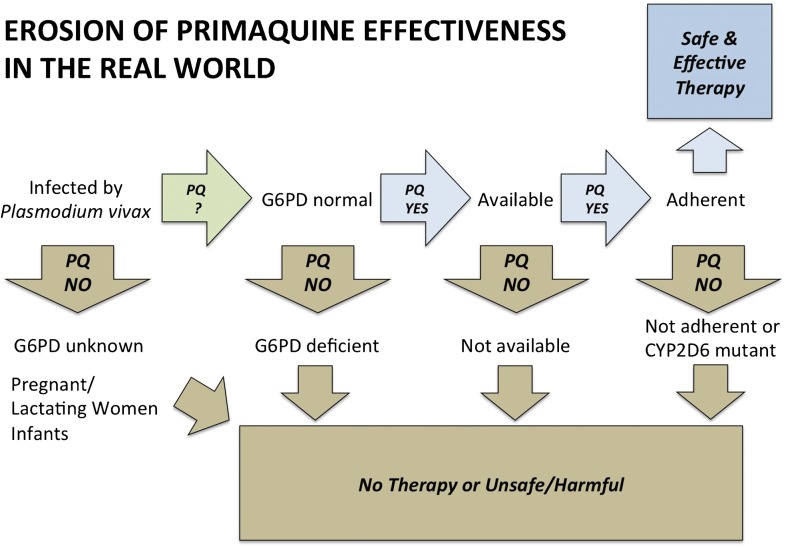
Diagram illustrating the step-wise loss of primaquine effectiveness in endemic settings.

Figure 2 illustrates the principle difficulties in delivering effective therapy for vivax malaria. The only drug effective against relapse of *P. vivax* is primaquine, a seriously flawed drug that causes mild to severe and fatal acute hemolytic anemia in patients having an inborn deficiency of glucose-6-phosphate dehydrogenase (G6PD).^17^ This very complex disorder affects 400 million people globally, with an average prevalence in malaria endemic zones of 8%.^18^ Current technologies do not permit access to a diagnosis of G6PD deficiency where most patients live. As a consequence of fear of causing harm, most providers either do not prescribe the treatment at all or fail to implore their patients to take the medication as directed. The 14 days of daily dosing with primaquine was designed with G6PD deficiency safety in mind, i.e. patients taking the drug under recommended clinical supervision could cease the regimen with onset of signs of hemolytic reaction. Not providing such supervision — by sending a patient home with stern instructions to complete the regimen — invites the danger of the patient ignoring or not noticing signs of hemolysis and steadfastly adhering to the doctor’s emphatic orders. Providers naturally temper such instructions, exacerbating the already problematic adherence issue with a 2-week regimen of dosing.

**Figure 3:**
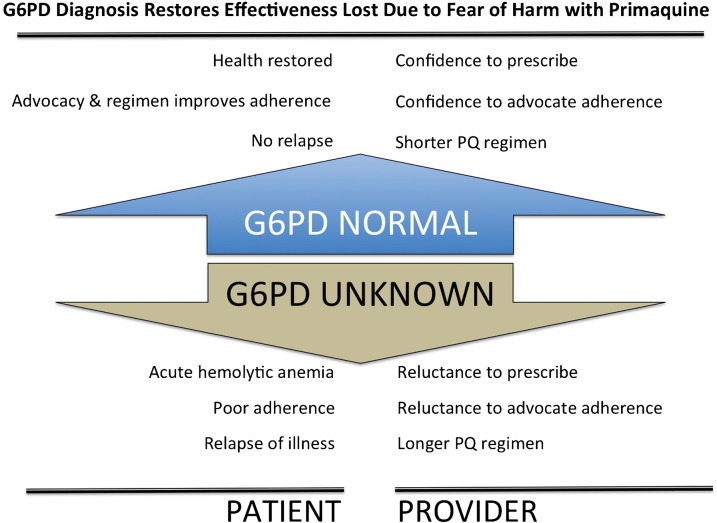
Diagram illustrating relief to ineffective primaquine therapy (among the G6PD normal majority) and its determinants and consequences contrasted with the benefits delivered by G6PD status ascertainment.

Most of these fear-related issues could be resolved with a point-of-care device that is practical and robust at the periphery of healthcare delivery where most malaria patients live.^19^ Figure 3 illustrates the many problems potentially solved by such a device.

Recent evidence has uncovered yet another potentially serious problem with primaquine — a mutation to cytochrome P-450 2D6 enzyme seriously impacts primaquine metabolism and degrades its efficacy against relapse.^20^ The frequency of mutant CYP2D6 among relatively extensively studied Caucasian populations ranged from 3% to 9%.^21^ If similarly high frequencies occur among the many diverse ethnic groups of Asia, those would define the proportion of patients in whom relapse cannot be prevented using primaquine.

## The Most Vulnerable — Impossible to Treat

Even assuming the availability of a robust point-of-care diagnostic device, proven safe and efficacious short dosing regimens of primaquine, and relatively low frequencies of CYP2D6 mutants (all optimizing effectiveness of the drug), there still remain very significant sub-populations that cannot be treated with primaquine. Unfortunately, these happen to be the same subpopulations most vulnerable to serious illness caused by vivax malaria — pregnant and lactating women, and infants.^22^–^25^ Primaquine cannot be prescribed to pregnant women due to the unknowable (as a practical rather than technical matter) G6PD status of the fetus. Most authorities recommend against primaquine during the first year of life, and some even exclude children up to the age of 4 years (WHO, for example^26^). Absent prolonged chemoprophylaxis or intermittent preventive therapy (untried strategies in this context), these patients will not receive primaquine and be subjected to repeated bouts of acute vivax malaria. Studies have demonstrated pregnant women, their fetuses, the infants borne to them, and the first year of life as all being relatively vulnerable to spontaneous abortion, stillbirth, and severe anemia.^22^–^25^ The prevention of infection in childbearing women and infants by means of minimizing exposure to biting anophelines should be considered a public health urgency in areas of transmission of vivax malaria.

## Preventable Harm

The successful treatment of vivax malaria in endemic zones requires overcoming a number of serious obstacles, and yet much depends on being able to do so. The failure to systematically attack the hypnozoite reservoir of *P. vivax*, which is the rule in most of the endemic world, results in repeated clinical attacks and serious illness, and further opportunities for transmission to others. Owing to the chronic neglect of this infection — largely driven by the erroneous supposition of its harmlessness — primaquine remains the only drug with which to combat that threat in the clinic. The conspicuous inadequacy of that drug, and the improbability of a rapid solution to it being grossly ineffective, should mobilize the global health community to re-examine measures aimed at minimizing mosquito–human contact, especially among the most vulnerable and untreatable (pregnant women and infants). Relatively aggressive measures are both indicated and likely achievable. These groups already represent the highest priority in programs distributing long-lasting insecticide-treated netting. Expanding such protection to treated curtains, indoor residual spraying of insecticides, spatial and topical repellants, and a variety of other personal protection measures ought to be strategized and implemented. For the time being, such represents the sole practical means of acting to mitigate the harm caused by vivax malaria.
